# Supplementary figures and images for: Proteomic Analysis of Mechanical Injury Effects in Papaya Fruit at Two Maturity Stages
Source: Proteomes. 2025 Sep 18;13(3):44. doi: 10.3390/proteomes13030044 (PMC12452570; doi:10.3390/proteomes13030044)

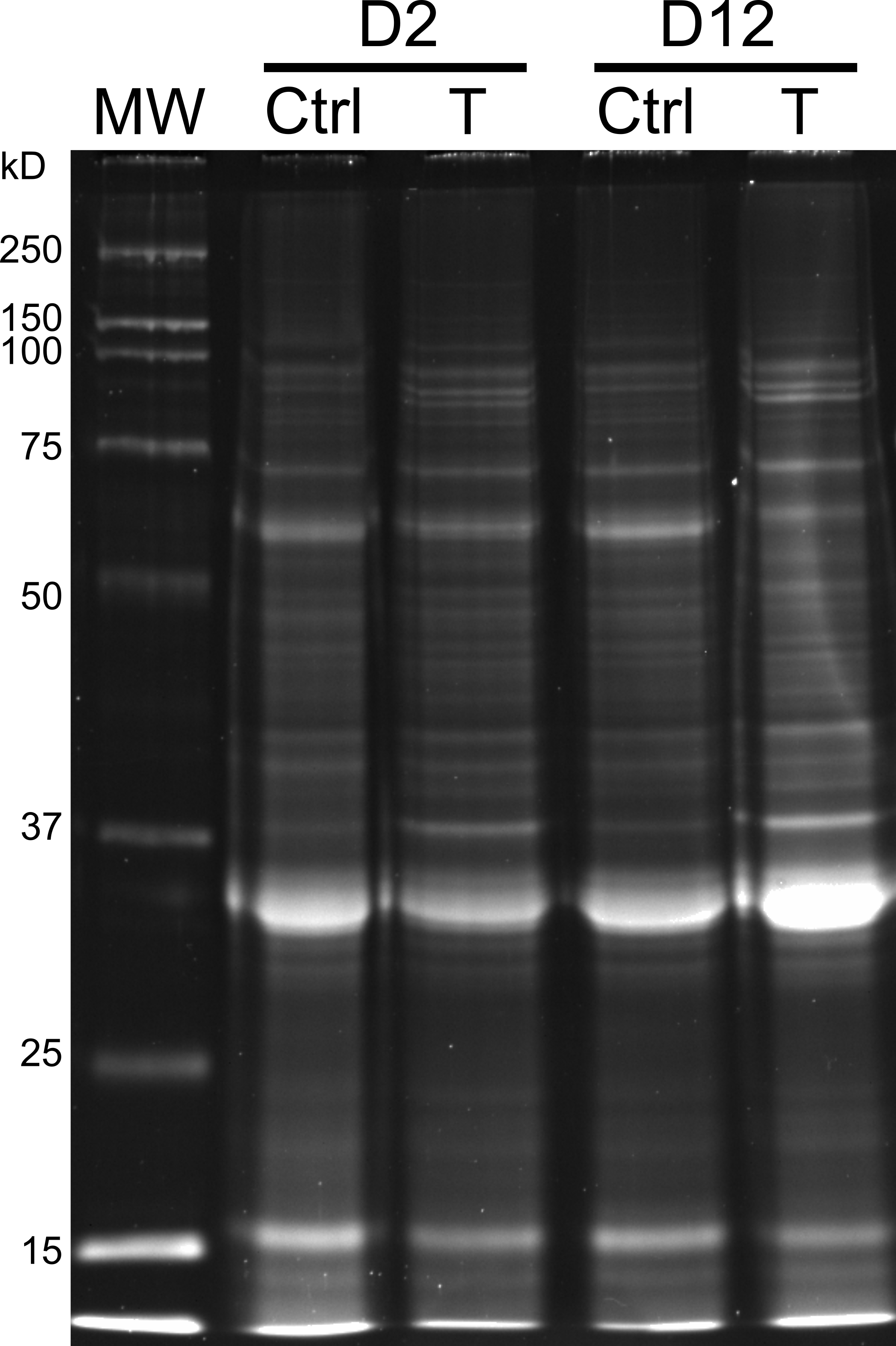

Supplement: Supplementary file 1 [file proteomes-13-00044-s001.zip › Figure S1.tiff]

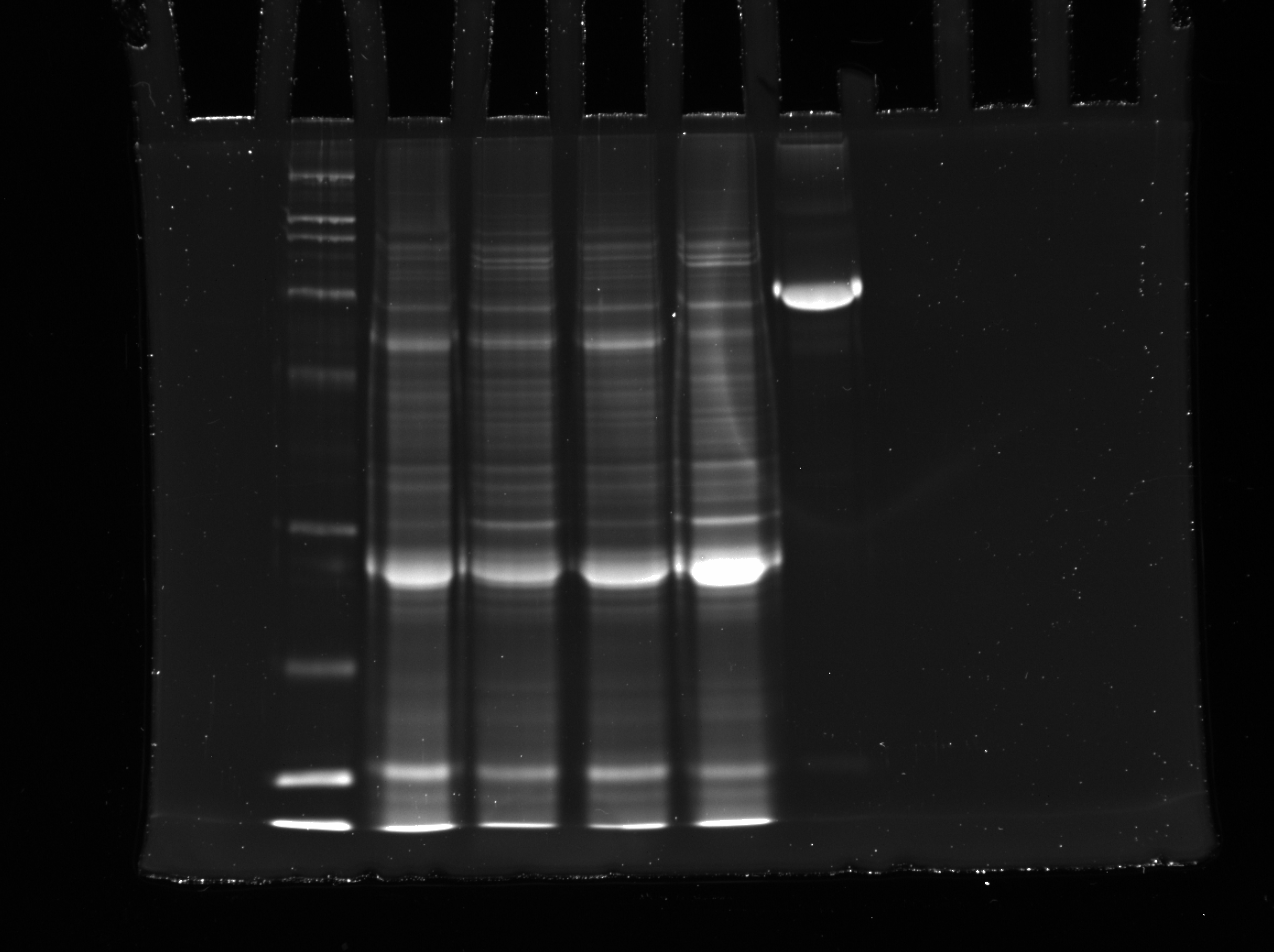

Supplement: Supplementary file 1 [file proteomes-13-00044-s001.zip › Figure S2.tif]
